# Editorial: Magnetoencephalography for social science

**DOI:** 10.3389/fnsys.2022.1105923

**Published:** 2023-01-04

**Authors:** Jonathan Levy, Iiro P. Jääskeläinen, Margot J. Taylor

**Affiliations:** ^1^Department of Neuroscience and Biomedical Engineering, Aalto University, Espoo, Finland; ^2^Ivcher School of Psychology, Reichman University, Herzliya, Israel; ^3^Departments of Medical Imaging and Psychology, University of Toronto, Toronto, ON, Canada; ^4^Diagnostic Imaging, Hospital for Sick Children, Toronto, ON, Canada

**Keywords:** magnetoencephalography (MEG), social neuroscience, social neurodevelopment, cognitive and affective neuroscience, functional connectivity

The past two decades have seen the growth of the field of social and affective neuroscience. By using a range of neuroimaging tools, research in this field has progressed in uncovering the neural substrates and systems involved in social and affective processes (Cacioppo and Decety, 2011; Singer, 2012; Stanley and Adolphs, 2013). Yet, despite half a century of invaluable contribution to various aspects in neuroscience, magnetoencephalography (MEG) (Hämäläinen et al., 1993; Baillet, 2017; Hari and Puce, 2017; Gross, 2019) has only recently been applied to investigating social and affective processes: From probing neural dynamics, through quantifying connectivity patterns of large-scale brain systems, to representing neural generators of multiple rhythms and oscillations and how they relate to social and affective behaviours. Examples range from studies on interpersonal interaction (Hari et al., 2015; Levy et al., 2017), developmental emotion inhibition (Vandewouw et al., 2021), watching social movies (Chang et al., 2015; Levy et al., 2016b), theory of mind (Vistoli et al., 2011; Mossad et al., 2016, 2021; Yuk et al., 2020); intergroup empathy (Levy et al., 2016a; Zhou et al., 2020), emotional face processing (Roesmann et al., 2020; Im et al., 2021; Safar et al., 2021), intergroup bias and interventions (Levy et al., 2021, 2022). These recent works revealed the potential of MEG in uncovering complex neural dynamics underlying psychological processes in the social world (Levy et al., 2020). In this special topic of “*Magnetoencephalography for social science*” we aimed at expanding this literature by editing four MEG studies that investigate various social processes: from morality, through the neural development of social-cognitive functioning and of face processing, to surprise. These studies, which are summarized below, relied on the uniqueness of MEG data to reveal complex neural dynamics underlying these social processes.

In one study, Hiraishi et al. used MEG to distinguish between morally good and bad judgments. They addressed a gap in the literature on the neuroscience of morality that has mainly focused on brain areas using fMRI neuroimaging, while rarely examining the temporal and connectivity patterns related to moral processes. Using MEG, the study revealed differences at regional, temporal and functional connectivity levels.

Another study by Sato et al. investigated a vulnerable population of children who were born with very low birth weight. They investigated their functional connectivity at rest and its associations with early nutrition and IQ and behavioural problems. The authors found that at preschool-age, these children show altered resting-state connectivity despite IQ and behaviour being in the average range, possibly reflecting functional reorganization of networks to support social-cognitive and behavioural functioning. The authors emphasized the importance of early postnatal nutrition in the development of resting-state networks that support social functions in childhood.

A paper by Mousavi et al. analyzed MEG data in an oddball task to investigate the phenomenon of surprise from a detailed neurodynamic outlook. Although surprise has been examined in many studies, the authors set forth an information-theoretical model to describe and predict the surprise level of an external stimulus in MEG data. The results of their analyses found that middle temporal components and the right and left fronto-central regions offer the strongest power for decoding surprise. The authors concluded that this is a practical and rigorous method for evaluating the interactive and social effects of surprising events on the brain.

Finally, Chen et al. studied face processing during the developmental period between 1 and 4 years. Despite this period being characterized by rapid changes in the ability to encode facial information, there are very few studies which investigate the neural processes of face encoding during this age, and this study sought to fill the gap between prior studies in infants and school-aged children. While implementing a longitudinal MEG approach across the first 4 years of life, they examined the maturation of the MEG responses in the fusiform gyri, which are primary nodes in the face-encoding network. The findings reported face-sensitive maturational changes, providing foundational data to the literature.

Overall, although MEG studies on social processes are increasing, the contribution of this Research Topic has been to consolidate this budding literature and increase exposure to this growing field of research. We contend that this has the potential to motivate social neuroscientists to use MEG, on one hand, and on the other hand motivate MEG scientists to explore social processes. Our ambition is to support the use of this non-invasive method for exploring new horizons that would expand the understanding of social phenomena and their underlying neural mechanisms. Finally, we note that recent technological progress in wearable-MEG, optically-pumped magnetometers, dual-MEG and multiple-brain analyses will certainly yield novel ecologically-valid paradigms that would enhance MEG's ability to emulate real-life validity in the near future (Hari et al., 2015; Brookes et al., 2022).

## Author contributions

JL wrote an initial draft for the editorial. All authors contributed to editorial revision, read, and approved the submitted version.
